# Evaluation of current methods to detect the mutations of epidermal growth factor receptor in non-small cell lung cancer patients

**DOI:** 10.1186/2049-6958-7-52

**Published:** 2012-12-11

**Authors:** Jasmina Obradovic, Vladimir Jurisic

**Affiliations:** 1 Institute of Biology and Ecology, Faculty of Science, University of Kragujevac, Radoja Domanovica 12, 34000, Kragujevac, Serbia; 2 Faculty of Medicine, University of Kragujevac, Svetozara Markovica 69, 34 000, Kragujevac, Serbia

**Keywords:** EGFR, Methods, Mutations, NSCLC

## Abstract

Many different methods were developed to detect commonly known mutations and to screen new mutations of the epidermal growth factor receptor in non-small cell lung cancer patients. Some of these methods are so sensitive as to be able to detect even one epidermal growth factor receptor mutant tumor cell among up to 1000–2000 normal cells. We have considered current methods chronologically reported to detect mutations in epidermal growth factor receptor in patients with non-small cell lung cancer. We also gave a short preview of their significance for routine clinical works. A Pub Med literature search was performed in order to demonstrate what methods are mostly used in mutation detection and to show their distribution through the last 10 years.

## Review

### Introduction

An extensive genetic research has provided a lot of useful information about molecular genetic abnormalities, including chromosomal aberrations, over-expression of oncogenes, and deletions or mutations in tumor suppressor genes. These results have been applied to early detection, classification, and prognosis of NSCLC [1].

Epidermal growth factor receptor (EGFR) is a trans-membrane receptor protein with a ligand-binding extracellular domain, trans-membrane domain, and cytoplasmic tyrosine kinase (TK) domain. EGFR is a member of a family of four tyrosine kinase receptor (RTK) molecules. Several ligands bind with receptor(s) and activate them inducing autophosporilation of TK domain, which is usually affected with mutations. This leads to a series of intracellular signaling pathways, which in turn result in cancer proliferation, reduced apoptosis, invasion, metastasis, and stimulation of tumor-induced angiogenesis [1].

Non-small cell lung cancer (NSCLC) is the most common cause of cancer-related death in the world [2]. EGFR is over-expressed in several tumor types, including NSCLC, and it was one of the molecules that were recognized as a biomarker for the development of targeted therapies [3,4]. The deletion of the four amino acid sequence (del 746–750) in the exon 19 and the substitution of leucine by arginine at codon 858 (L858R) in exon 21 are two of the most common mutations in the kinase domain of EGFR gene in NSCLC patients [5].

The small-molecule tyrosine kinase inhibitors including gefitinib and erlotinib have recently been approved for the treatment of patients with NSCLC [4,6-9]. In addition, mutations in the epidermal growth factor receptor (EGFR) have been confirmed as predictors of the efficacy of treatment with EGFR-tyrosine kinase inhibitors. The results from several randomized phase III trials have emphasized the importance of molecular testing prior to initiating first-line therapy for advanced NSCLC. Increasing evidence demonstrates that patients with EGFR mutations experience a more significant benefit with gefitinib or erlotinib compared to standard chemotherapy, whereas an opposite effect occurred in patients with EGFR-mutation negative tumors [7-9].

We have considered several methods to detect EGFR mutation reported in literature and come into use in the last decade. In addition we have analyzed a variety of sophisticated novel methods and previewed their significance for routine laboratory and clinical work.

### Data review and interpretation

**Figure 1:**
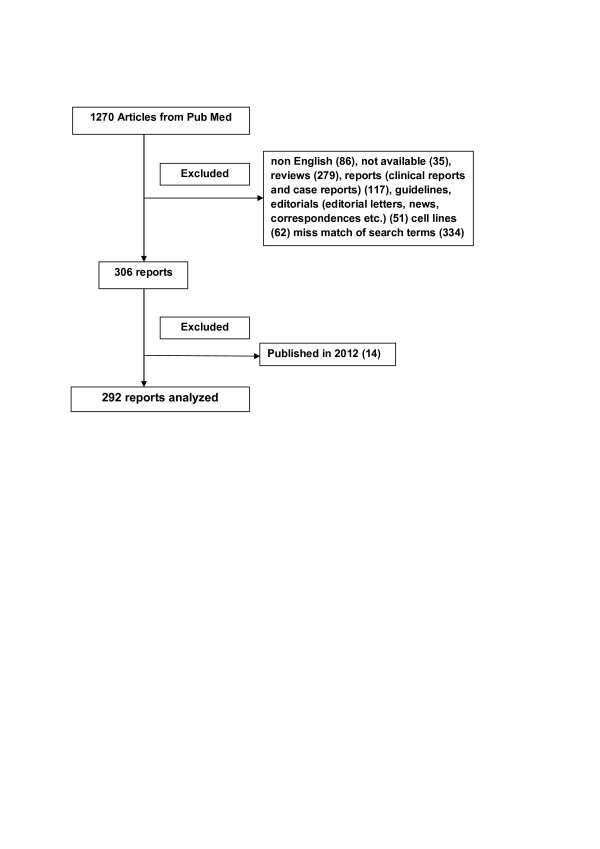
Diagram of included and excluded articles (292 articles were chosen for analysis).

Pub Med literature was reviewed in May and April 2012 for all studies published from Jan 1, 2000 to December 31, 2011. In this search we took next key words: “mutations”, “epidermal growth factor receptor”, “EGFR”, “non-small cell lung cancer”, “NSCLC”, actually “mutations epidermal growth factor receptor EGFR non-small cell lung cancer NSCLC” in single search, with activated limits that included “Humans”, “Cancer” and “Publication Date”. We thus obtained 1,270 articles. Articles included in our analysis are presented in Figure 1.

We excluded reviews, reports (clinical trials, and case reports), guidelines, editorials (editorial letters, news, correspondences etc.), reports that were not in English language or were not available, those which mismatched our search terms (articles that discussed about other proteins of mentioned signal pathways, not about EGFR, or about other diseases) and articles published in 2012 (Figure 1).

### Overview of current methods for detection mutations of EGFR in NSCLC patients

#### Immunohistochemistry

Immunohistochemistry (IHC) is based on the binding of antibodies tagged with a visible label to the specific antigens in tissue sections [10]. Immunohistochemical analyses are routinely performed in clinical laboratories and they can simultaneously analyze expression level of proteins or protein modifications. The advantage of this technique for a wide use in clinical practice is the possibility to preserve tumor morphology [11]. Immunohistochemical method with monoclonal antibodies is much easier and more cost-effective than analysis of extracted DNA [12].

#### FISH assay (fluorescence in situ hybridization)

This is a method for detection of number of copies of specific genes and location of the target sequences by fluorescent label probe [13]. The main barriers to routinely performing FISH in clinical laboratories include lack of availability in molecular techniques and of experience with the equipment for the dark-field fluorescence microscopy that is needed to assess copy number [11,14].

One alternative to FISH might be chromogenic *in situ* hybridization (CISH), which uses bright-field light microscopic techniques to assess gene copy number and seems to be accurate and reproducible at the same time [11,15].

#### High-performance liquid chromatography (HPLC)

Denaturing high performance liquid chromatography (DHPLC) is another method to detect somatic and inherited mutations [16]. NSCLC specimens were analyzed by high performance liquid chromatography on the Transgenomic WAVE HS system by Jänne [17]. It is a rapid method for EGFR mutation screening with 100% sensitivity and without false negatives. It could detect clinically relevant mutations in small diagnostic specimens. Mutations in exons 18 to 21 of EGFR were analyzed using a DNA endonuclease, SURVEYOR, which cleaved mismatched heteroduplexed DNA. For these analyses DNA could be prepared from both frozen and formalin-fixed, paraffin-embedded (FFPE) tumor specimens, without micro or gross dissection. This scanning technique is superior to direct sequencing when used with undissected formalin-fixed, paraffin-embedded specimens. Seven out of 160 (4%) mutations not previously detected by direct sequencing could be detected using SURVEYOR [17].

Cohen found advantages of DHPLC [18], like detection of mutations in exons 19 and 21, without resorting to the digestion step described in the Jänne et al., which makes this method less costly. Genotyping with this technique can be done very rapidly. However, homozygous mutation would miss this method and DHPLC lacks the capacity to predict the exact nature of the mutation. Therefore, subsequent verification by direct sequencing would still be needed to identify the mutation in positive cases [18].

Tan Min Chin developed a partially denaturing HPLC (pDHPLC) assay [19] to detect a large range of sequence variants with high sensitivity and low detection limits for minority alleles in an inexpensive and standardized manner. It is suggested as a useful approach for routine detection of EGFR variants [19].

#### DNA sequencing

One of the sequencing methods, known as direct DNA sequencing, is based on DNA synthesis *in vitro* having four precursor nucleotides (NTP’s) and four deoxynucleotides (ddNTP’s), one of which is often labeled in four polymerase reactions [20]. It has now been developed an automated sequencing and reactions are performed in a single tube containing all four ddNTP's, each labeled with a different color dye [21,22]. Also there are novel methods of sequencing like pyrosequencing [23,24], hybridization sequencing [25], and sequencing by denaturation [26].

Direct DNA sequencing of PCR-amplified genomic DNA has been developed to detect EGFR mutations in patient’s tumor tissue. Sensitivity of DNA sequencing is affected with some steps. Biopsy treatment usually provides small amount of tissue, which is often not enough amount of DNA for extraction from tumor samples. Paraffinisation of tissues after biopsies is another step where DNA could be lost, or it is a poor-quality for DNA sequencing regarding cross-contamination with DNA from stromal cells. The quality of this method is affected by the percentage of tumor cells in the sample and within the mutation. Routine use of this method in clinical laboratories is still often limited by financial and technical constraints and also by length of the procedure. It requires a few days to obtain a result after tissue acquisition [15,27]. This method involves multiple steps (DNA extraction, PCR-based amplification, DNA sequencing, and sequence interpretation). Sensitivity of direct sequencing is suboptimal for clinical tumor samples; mutant DNA needs to comprise ≥25% of the total DNA to be easily detected [9].

##### Polymerase chain reaction

The polymerase chain reaction (PCR) is a rapid method used for exponential amplification of a particular DNA sequence. PCR can be extensively modified, and has a wide range of applications [28,29]. We describe here some usually applied PCR techniques to diagnose EGFR mutations in NSCLC.

Reverse Transcription PCR (RT-PCR) is a method that uses RNA as a template for an enzyme, reverse transcriptase that transforms RNA into cDNA which is then amplified by regular PCR. RT-PCR is widely used to determine the expression of a gene and does not require post-PCR sample handling, preventing potential PCR product contamination and resulting in much faster and higher throughput assays [30,31].

Quantitative PCR(qPCR, also called real-time PCR) is precisely a method used to measure the quantity of a PCR product in a real-time. It is used to determine the presence of a DNA sequence in a sample and the number of its copies in the sample. This qPCR uses fluorescent dyes, such as Sybr Green, EvaGreen or fluorophore-containing DNA probes, such as TaqMan, to measure the amount of amplified product in real time [32,33].

##### The mutant-enriched PCR

The mutant-enriched PCR is a rapid and sensitive assay and it can detect one mutant gene among as many as 10^3^ to 10^4^ copies of the wild-type gene [34-36]. It eliminates wild-type genes selectively and enriches the mutated genes, in two-step PCR with intermittent restriction digestion [37].

This method can detect EGFR mutations in various kinds of clinical samples including specimens by biopsies, pleural fluid, and surgically resected tissues from patients with NSCLC. But this assay can only be used to analyze specific alterations containing a commonly deleted region of exon 19 (codons 747–749) and the L858R mutation of exon 21. It cannot detect minor alterations, like mutations in exon 18, minor deletions of exon 19, and exon 20 insertions [37]. False-positive result could occur within the mutant-enriched PCR caused by high PCR cycle number. It caused replacement of a critical nucleotide. Restriction site of wild-type fragments could be destroyed. Results were confirmed with the mutant-enriched PCR assay at least twice and also performed sequencing to confirm the EGFR alterations. This is an important and sensitive assay, because it can detect commonly known mutations in heterogeneous clinical samples that may contain a small fraction of mutated genes and a large amount of wild-type genes [37].

Several data indicated that methods of mutant-enriched PCR could be used in pleural effusion for screening EGFR mutation in inoperable advanced NSCLC patients. The results of comparing direct sequencing with mutant-enriched PCR indicated that a significant portion of mutations could be missed by using direct sequencing [38].

##### Peptide nucleic acid-locked nucleic acid (PNA-LNA) PCR clamp

PNA-LNA PCR clamp is a sensitive and rapid method that was used for simultaneous detection of 11 different EGFR mutations [39]. In addition, several authors [40] screened about 30 non–small cell lung cancer cell lines, and among these established cell lines from Japan people’s, eleven showed mutations. Briefly, these results indicated that many cell lines have subpopulations that hold specific EGFR mutations [40].

When using PNA clamp primers, amplification of the wild-type sequences is suppressed, but amplification of the mutant sequences is enhanced. LNA probes specifically detect mutant sequences in the presence of wild-type sequences. Because PNA clamp primers have wild-type sequences and LNA probes have mutant sequences, they are located in the position. PNA clamp primers competitively inhibit mutant LNA probes to bind to the wild type, further increasing the specificity of detection. Thus, individual EGFR mutations can be detected in the presence of 100- to 1,000-fold wild-type EGFR background molecules. Researchers were multiplexed the reactions by using multiple probes labeled with different dyes to detect 11 mutations by five reactions. Even after being multiplexed, each mutation was detected in the presence of 100- to 1,000-fold background [40].

##### Double-stranded PCR product formation assay performed by Light Cycler

This assay was performed by Sasaki [41] who investigate EGFR mutation status in Japanese lung cancer patients. The principle of this method is measuring fluorescence of the SYBR Green dye when it is intercalated in double-stranded DNA, during PCR reaction, so it detects double-stranded PCR product formation [41].

For the detection of mutations in very small amounts of DNA is often used “nested PCR”, that is avoided by using this assay. The advantage of this method is avoiding ethidium bromide stain, which is potentially toxic reagent. Researchers developed three different PCRs to detect EGFR gene mutations and deletions. Three common EGFR mutations were analyzed, (in exon 18, a G719S mutation, deletion in exon 19 and (CTG → CGG; L858R) mutation in exon 21) by real-time quantitative PCR with mutation-specific sensor and anchor probes [41].

Although researchers have only checked the three most frequent mutations, this is a rapid method without need for any post-PCR sample manipulation, which saves time and minimizes the risks of DNA contamination. Double-stranded PCR product formation assay performed by Light Cycleris is proposed for screening and treatment evaluation, so it could be used in predicting the sensitivity or resistance to TKI therapy for lung cancer patients [41].

##### Polymerase chain reaction single-strand conformation polymorphism (PCR-SSCP)

In a study performed by Marchetti [42], PCR-SSCP analysis was found to be more sensitive than direct sequencing of PCR products, allowing identification of mutations that were hardly detectable or undetectable (21% of cases) by direct sequencing. Mutations missed by direct sequencing were all point mutations. It was examined in NSCLC patientsfor EGFR mutations in exons 18, 19, and 21 using a direct sequencing of polymerase chain reaction products and PCR-SSCP analysis. More than 90% of the mutations in the EGFR gene could be immediately recognized by SSCP analysis. No false-positive or false-negative results were obtained using the SSCP assay. PCR-SSCP assay could be performed on formalin-fixed, paraffin-embedded small biopsies [43], allowing the detection of mutations from minimal amounts (<1 μg) of starting DNA. The authors found three new mutations (two new types of deletions and a new amino acid substitution at codon 858) and confirmed the presence of several hot spot mutations [42].

#### Frequency of current methods reported in literature for EGFR detection

**Figure 2:**
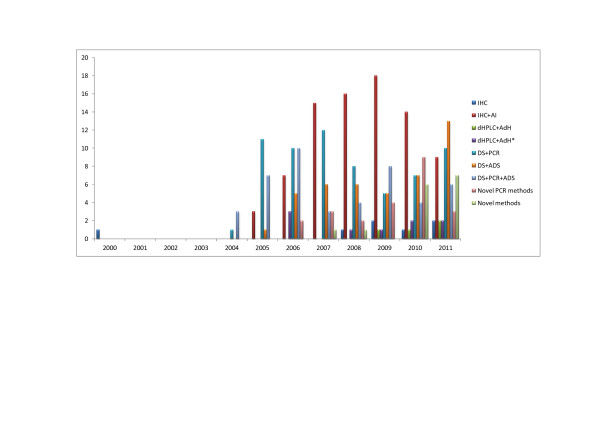
Numbers of published articles and methods to detect mutations of EGFR in NSCLC patients through the years.

**Figure 3:**
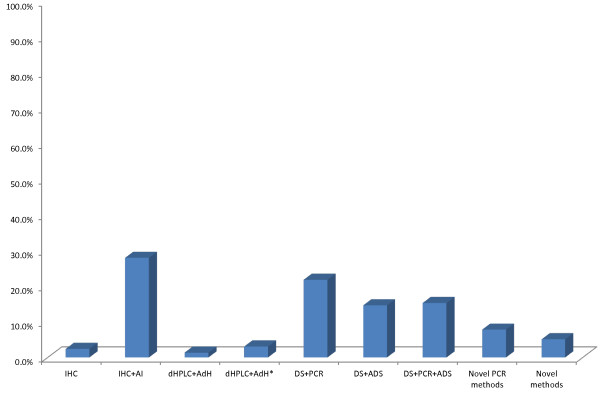
Frequency of the methods used in the total selected period (2000–2011 years).

We have presented numbers of published articles and methods that were used for the set period of time (Figures 2 and 3). Immunohistochemical analyses (IHC), were not used only as single methods but very often in combination with direct sequencing (DS), and/or fluorescence in situ hybridization (FISH) and/or chromogenic *in situ* hybridization (CISH). Also these combinations involved peptide nucleic acid-locked nucleic acid (PNA-LNA) PCR clamp for EGFR mutation analyses. Denaturing high performance liquid chromatography (dHPLC) was analyzed with direct sequencing, or even with some additional methods like PCR, Scorpion Amplified Refractory Mutation System technology (SARMS) [44].

Search criteria that included “polymerase chain reaction” referred also to real time polymerase chain reaction (or quantitative polymerase chain reaction) as well as reverse transcription polymerase chain reaction. Among novel PCR methods there are methods like: cycleave real-time PCR [45], Scorpion Amplified Refractory Mutation System technology (SARMS) [46] or nano-fluidic digital PCR arrays [47]. As novel methods for estimation of EGFR have also been reported: fully automated system with a nano-scale engineered biomagnetite [48], colorimetric detection of mutations in EGFR using gold nano-particle aggregation [49] or detection of the EGFR mutation in NSCLC using molecular beacons [50].

The distribution of methods used in ERGF mutation detection for the set period of time is illustrated in Figure 2. Immunohistochemical analyses in combination with PCR, direct sequencing, FISH or CISH were more frequently used in the period from 2001 to 2005. The immunohistochemistry is probably mostly used in clinical practice based on the consideration that protein expression level can be evaluated directly on tumor cells or on other specific cells of interest from formalin fixed and paraffin embedded tissue. However, some subjectivism in visual description contributes to the limitation of this semiquantitative method [51]. The main lack of FISH technology is an epifluorescent system, that could be overcame with CISH which uses bright-field microscopy [51].

DNA sequencing, in the majority of the research paper confirmed data analyzed previously by any other methods. The advantage of PCR methods is that they enable the analysis of a small tissue samples, like cells from body fluids, bronchial washings, fine needle aspirates, as well as circulating tumor cells.

DHPLC is used nowadays but often with direct sequencing. This method showed higher sensitivity when compared to gene sequencing for most frequent mutations of EGFR (deletion mutation and L858R mutation). It was shown that the frequency of these mutations detected by DHPLC was 30.1%, and that the analyses were technically easier and less expensive for routine clinical practice [44].

In the past six years direct sequencing, in combination with PCR alone or with PCR and other advanced techniques, has widely been used to detect EGFR mutation in NSCLC. Recently, sensitive approaches to detect mutation have been developed, including advanced PCR methods, but they are less applied. PCR methods in combination with highly sensitive methods usually need confirmation by direct sequencing, but they allow modifications and specialization, so it could be said they are almost inevitable.

There are several differences in interpreting the intensity of expression and the localization of receptors, and the wide range of methods in use for EGFR detection, that causes heterogeneity of available reports [52]. So, there is no definitive conclusion and recommendation on which are the best methods to detect the mutations of EGFR in NSCLC patients. Through the analysis of the literature we gathered some problems that might be helpful in the choice of methods to use: heterogeneity of the samples for analysis [15,37]*;* different types of ligands that activate EGFR, and other members of epidermal growth factor receptor family; plenty of signal downstream pathways after receptors activation [53]; development of resistance after TKIs treatment and appearance of secondary mutations [54]; undefined and unclear relation between EGFR over-expression and tumor invasiveness [55,56]; adverse effects of chemotherapy [57]; polymorphism of EGFR gene [58]. Non membrane bound events and other mechanisms of increased signaling in the modulation of specific behaviors should also be considered [52]. There are certain efforts to provide universal scoring system and standardization of techniques and this is discussed by Molecular Assays in NSCLC Working Group. They recommend EGFR molecular assays for the use and propose guidelines for tissue storage, handling, and processing [11].

## Conclusion

A lot of methods are used to determine mutations of EGFR. These methods described here are highly sensitive, but usually available to detect only commonly known mutations of EGFR in NSCLC patients. DNA sequencing is widely used, but it is time-consuming and sensitivity of this method is often concerned.

We wish to point out that certain methods described here are generally used in the diagnosis of the tumor from biopsy. Other more sensitive methods are recommended to determine the presence of the mutation of EGFR from small tissue quantities. EGFR gene copy number can be assessed by a variety of methods, including FISH, CISH, and real-time quantitative PCR. Several publications reported up to 90% sensitivity for detection of EGFR mutations in circulating tumor cells isolated from patients with metastatic NSCLC [47,48].

However, to detect the presence of minimal residual disease or molecular disease remission – thus solving a big problem in the clinical practice - we would suggest the need to introduce new, highly specific and more sensitive methods constantly.

## Abbreviations

AdH: Additional methods to dHPLC, like DS; AdH*: Additional methods to dHPLC: one or more than one; ADS: Additional methods to DS: one or more than one; AI: Additional methods to IHC, like: and/or D.S + and/or PCR + and/or fluorescence in situ hybridization (FISH) + and/or chromogenic in situ hybridization (CISH); dHPLC: Denaturing high performance liquid chromatography; DS: Direct DNA sequencing; IHC: Immunohistochemical analysis; PCR: Polymerase chain reaction.

## Competing interest

The authors declare that they have no competing interests.
